# A Toll-like Receptor-Activating, Self-Adjuvant Glycan Nanocarrier

**DOI:** 10.3389/fchem.2022.864206

**Published:** 2022-05-03

**Authors:** Daping Xie, Yiming Niu, Ruoyu Mu, Senio Campos de Souza, Xiaoyu Yin, Lei Dong, Chunming Wang

**Affiliations:** ^1^ State Key Laboratory of Quality Research in Chinese Medicine, Institute of Chinese Medical Sciences, University of Macau, Taipa, Macao SAR, China; ^2^ State Key Laboratory of Pharmaceutical Biotechnology, School of Life Sciences, Nanjing University, Nanjing, China

**Keywords:** polysaccharides, toll-like receptors, self-adjuvant, glucomannan, vaccination

## Abstract

The global pandemic of COVID-19 highlights the importance of vaccination, which remains the most efficient measure against many diseases. Despite the progress in vaccine design, concerns with suboptimal antigen immunogenicity and delivery efficiency prevail. Self-adjuvant carriers–vehicles that can simultaneously deliver antigens and act as adjuvants–may improve efficacies in these aspects. Here, we developed a self-adjuvant carrier based on an acetyl glucomannan (acGM), which can activate toll-like receptor 2 (TLR2) and encapsulate the model antigen ovalbumin (OVA) via a double-emulsion process. *In vitro* tests showed that these OVA@acGM-8k nanoparticles (NPs) enhanced cellular uptake and activated TLR2 on the surface of dendritic cells (DCs), with increased expression of co-stimulatory molecules (e.g. CD80 and CD86) and pro-inflammatory cytokines (e.g. TNF-α and IL12p70). *In vivo* experiments in mice demonstrated that OVA@acGM-8k NPs accumulated in the lymph nodes and promoted DCs’ maturation. The immunization also boosted the humoral and cellular immune responses. Our findings suggest that this self-adjuvant polysaccharide carrier could be a promising approach for vaccine development.

## Introduction

The global calls for COVID-19 vaccination remind us that vaccines remain the most established interventions against numerous infectious diseases in human history (Tregoning et al., 2021). Elevating antigens’ immunogenicity and delivery efficiency is the central goal for modern vaccine design (Saso and Kampmann 2017), and adjuvants are designed to perform such roles. Classical adjuvants, including aluminium salts (Alving et al., 2012) and Montanide ISA 51 (van Doorn et al., 2016), enhance antigen delivery to antigen-presenting cells (APCs), enable sustained release and uptake of the antigen, and cause inflammation that benefits immunocytes accumulation (Lambrecht et al., 2009), but their mechanisms remain elusive and specificity varies. For instance, aluminum salts hardly induce cytotoxic T cell response on their own; their adsorbed proteins are usually less stable than in the solution and their enhancing function is often restricted to whole-cell antigens (Sivakumar et al., 2011). Modern adjuvants have relatively specific biological activities. Some of them target pattern-recognition receptors (PRRs) expressed on the surface of APCs, such as toll-like receptor 4 (TLR4), which can be activated by specific ligands like monophosphoryl-lipid A (MPLA) (Mata-Haro et al., 2007). However, these adjuvants are simply mixed with antigens for potential clinical uses, leading to antigen-adjuvant dissociation *in vivo* (Wang and Xu 2020), off-target delivery, and weakened efficacy (Apostólico et al., 2016). Therefore, to solve this problem, the abovementioned two types of adjuvant can be combined in use, such as AS04 composed of MPLA adsorbed on aluminium hydroxide (Garçon et al., 2017), but a simple mixture of two components with distinct physical properties (e.g., water solubility) brings about technical concerns over the stability, bioavailability and sterilization method (Garçon and Di Pasquale 2017). Integrating both the physical function (as a vehicle to deliver) and biological activity (as a PRR agonist to boost) in one adjuvant—“self-adjuvant carrier”—has attracted considerable attention.

Polysaccharides have unique advantages as the material source for designing self-adjuvant carriers, thanks to their physiological abundance (Mishra et al., 2016), good biocompatibility (Rogovina et al., 2018) and, most desirably, immune activities. Physically, polysaccharides are commonly used as polymeric vehicles for drug delivery and tissue engineering. Biologically, immune cells recognize a diverse range of carbohydrate signals. On the surface of many bacterial and fungal cells exist carbohydrate signals, known as pathogen-associated molecular patterns (PAMPs). These sugars can activate multiple PRRs on innate immune cells (Sun et al., 2019), notably including macrophages and dendritic cells (DCs) (Niu et al., 2017), stimulating these cells to produce cytokines that in turn boost immune responses. For instance, yeast zymosan triggers inflammatory responses through activating TLR2 and dectin-1 (Underhill, 2003); bacterial lipopolysaccharide (LPS) exerts robust activities via TLR4 (Mata-Haro et al., 2007). Polysaccharides from non-microbial origins can also mimic such actions. In our previous work, inspired by microbial signals, we found that acetylation of hydroxyl groups to a certain degree enables plant-derived glucomannan (GM) to activate TLR2 on macrophages with little toxicity in mice (Feng et al., 2019). TLR2 is a major member of the TLR family. Following ligand stimulation, TLR2 forms heterodimers with TLR1 or TLR6 to initiate a cascade leading to robust innate immune responses, activating pro-inflammatory transcription factors (e.g., NF-κB and AP-1) to produce cytokines (Piao et al., 2016). In addition, activating TLR2 could increase the expression of co-stimulatory molecules, such as CD40 and CD80/86 on the surface of DCs (Martínez et al., 2020). Acetyl GM (acGM) has the potential to serve as a new source for adjuvant design.

**FIGURE 1:**
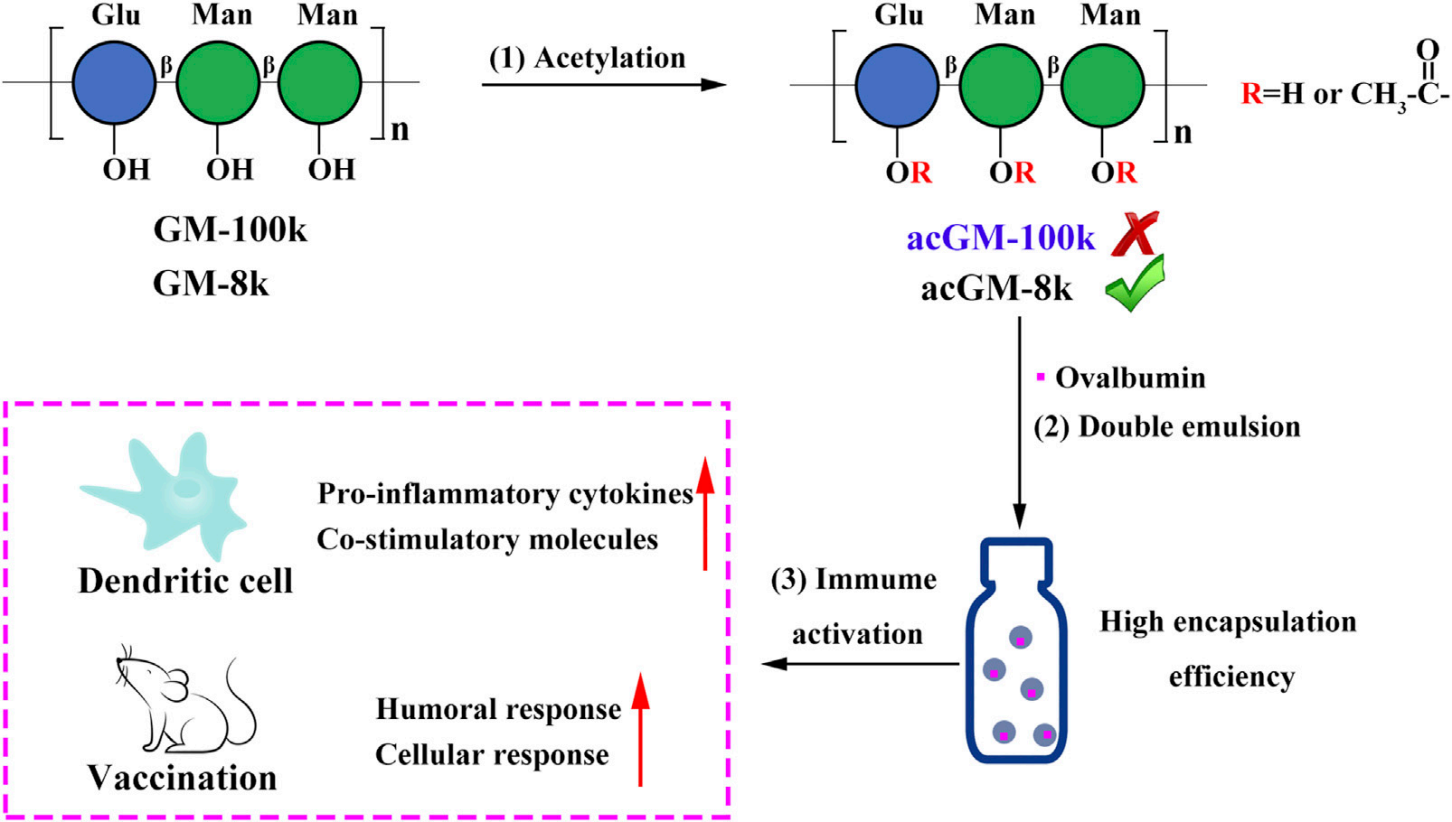
Schematic illustration of designing a self-adjuvant carrier for antigen delivery. (1) Acetylation of glucomannans (GM) with different molecular weights (100 and 8 kDa). (2) Encapsulation of ovalbumin (OVA) into acetyl GM (acGM) through a double-emulsion method. (3) Evaluation of the OVA-loaded acGM-8k NPs (OVA@acGM-8k NPs) in promoting the humoral and cellular immunity in mice.

In this study, we aimed to develop acGM into a self-adjuvant carrier. We preferred encapsulating the antigen in–rather than mixing it with–acGM, because encapsulation protects antigen from degradation (Pati et al., 2018) and promotes the cellular uptake of antigen (Mitchell et al., 2021). To realize this design, we optimized the physical characteristics of the linear polymer of acGM in association with its capacity to encapsulate antigens. We prepared GM with molecular weights of approximately 100 and 8 kDa and subsequently acGM-100k and acGM-8k with a degree of substitution (DS) of 1.8, followed by a double-emulsion procedure to fabricate nanoparticles (NPs) to encapsulate the model antigen ovalbumin (OVA) (Figure 1). We tested the immunizing performance of this adjuvant system *in vitro* and *in vivo*.

## Materials and Methods

### Chemicals and Reagents

GM from Konjac was purchased from Shimizu Chemical Corporation. Pam_3_CSK_4_, Normocin™, Zeocin™, HECK-Blue™ selection were purchased from Invivogen. Fetal bovine serum, RPMI 1640 medium and DEME medium were purchased from Gibco. OVA and penicillin-streptomycin (100×) were purchased from Sigma-Aldrich. All other chemicals and reagents were purchased from Aladdin.

### Cell and Animals

HEK-Blue™ mTLR2 and HEK-Blue™ Null2 cells were purchased from Invivogen and cultured in DMEM medium supplemented with 10% fetal bovine serum and 1% penicillin-streptomycin. DC2.4 cells were purchased from ATCC and cultured in RPMI 1640 medium supplemented with 10% fetal bovine serum and 1% penicillin-streptomycin. Bone marrow-derived dendritic cells (BMDCs) were isolated from 6 to 8 weeks C57BL/6J mice and cultured in RPMI 1640 medium supplemented with 10% inactivated fetal bovine serum and 1% penicillin-streptomycin. 20 ng/mL GM-CSF and 10 ng/ml IL4 was used for BMDC differentiation.

### Synthesis and Characterization of acGM-100k and acGM-8k

acGM-100k was synthesized according to our previous work starting from high molecular weight GM (about 100 kDa) (Feng et al., 2019; Mu et al., 2021). acGM-8k was synthesized starting from low molecular weight GM (about 8 kDa). Briefly, 1.0 g GM was dissolved in 50 ml DMF stirred at 50°C for 1 h. Then 5.25 ml acetic anhydride (3 equivalent) and 4.591 ml pyridine (3 equivalent) were added. After 10 h, the mixture was poured into 100 ml dd-H_2_O and acGM-8k was extracted by dichloromethane. The extraction was washed with 100 ml 1 M HCl twice and 100 ml dd-H_2_O. The acGM-8k was obtained by evaporation as a pale-yellow powder. For acetylation by acetic chloride, 3.95 ml acetic chloride (3 equivalent) and 4.591 ml pyridine (3 equivalent) was added. Considering that the solubility varies with the change of DS, when studying the effect of reaction time on the DS, the product was obtained by dialysis and lyophilization. The acetyl dextran (acDEX) was synthesized using the same method.

### Preparation and Characterization of acGM-8k NPs and OVA@acGM-8k NPs

100 mg acGM-8k was dissolved in 1 ml dichloromethane. 100 μl PBS or PBS containing 5 mg OVA was added into the organic solution. This mixture was then emulsified by sonicating for 30 s on ice using a probe sonicator with an output of 20%. Then the primary emulsion was added to 2 ml 3% PVA solution and emulsified for another 1 min. The W/O/W was transferred to 20 ml 0.3% PVA solution and stirred to evaporate the dichloromethane. The nanoparticles were collected via centrifugation at a speed of 15,000 rpm for 15 min. Finally, the nanoparticles were re-suspended in 2 ml dd-H_2_O and lyophilized. The morphology of acGM-8k NPs was characterized by scanning electron microscopy with a S-5000 microscope after sputter coating with a palladium/gold alloy. Particle size distributions and zeta potential were determined by dynamic light scattering using a Zetasizer (Malvern Instruments). Particles were suspended in dd-H_2_O at a concentration of 2 mg/ml acDEX NPs were prepared using the same method.

### Loading Capacity and Encapsulation Efficiency

10 mg OVA@acGM-8k NPs were dissolved in 1 ml acetone and incubated at 37°C for 4 h. The precipitated OVA was collected by centrifuging at a speed of 15,000 rpm for 15 min. The pellet was then dissolved in 1% SDS solution. The content of OVA was determined by using fluorescamine method.

### *In vitro* Activation of BMDCs

BMDCs were isolated from 6 to 8 weeks C57BL/6J mice or TLR2 knockout mice with C57BL/6J background. The GM-CSF and IL-4-induced BMDCs were harvested at day 7. The BMDCs were incubated with acGM-8k NPs (100 μg/ml), acDEX NPs (100 μg/ml) or Pam_3_CSK_4_(100 ng/ml) for 24 h. The supernatant was discarded and the co-stimutory molecules were detected by flow cytometry. CD11c was used as the marker of BMDCs, CD80/86 and CD40 were used as the marker of matured BMDCs.

### Cellular Uptake of OVA@acGM-8k NPs

To investigate the intracellular trafficking of OVA@acGM-8k NPs, DC2.4 cells were incubated with OVA, OVA@acGM-8k NPs and OVA@acDEX NPs. After 2 h incubation, the cellular uptake was determined by flow cytometry. For confocal laser imaging, the DC2.4 was replaced with fresh medium and incubated for another 1, 3, 6 h. Then the DC2.4 cells were stained with Hoechst 33,342 for 15 min and lysotracker red for 15 min and visualized in Leica TCS SP8 Confocal Laser Scanning Microscope System.

### Cytokine Analysis

BMDCs (10^6^ cells per well, 6 well plate) were incubated with acGM-8k NPs, acDEX NPs (100 μg/ml) or Pam_3_CSK_4_ for 24 h the supernatant was collected and the level of pro-inflammatory TNF-α, IL12-p70, IL-1β and IFN-γ were determined using ELISA kit (Invivogen).

### Lymph Node Distribution of OVA@acGM-8k NPs and Activation of Lymph Node-resident DC *in vivo*

To investigate the distribution of OVA@acGM-8k NPs, 10 μg Cy5-OVA or 200 μg OVA@acGM-8k NPs, OVA@acDEX NPs containing 10 μg Cy5-OVA was injected subcutaneously into the footpad of mice. The lymph nodes (LNs) were visualized using an IVIS Spectrum system at designed time points. After 36 h, the popliteal LNs were isolated for *ex-vivo* imaging. The fluorescence intensity of the popliteal LNs was semi-quantified.

To investigate the activation of lymph node-resident DC *in vivo*, OVA@acGM-8k NPs were injected subcutaneously into the footpad of the mice. At 72 h, the popliteal LNs were isolated and homogenized into a single-cell suspension. The obtained cells were analyzed by flow cytometer.

### Animal Immunization and OVA-specific Antibody Detection

All immunizations were administered through hind footpad subcutaneous injection using an insulin syringe needle. Each formulation contained 10 μg of OVA or 200 μg of OVA@acGM-8k NPs containing 10 μg of OVA. After primary immunization on day 0, booster immunizations were given on day 14. Blood samples were obtained from the angular vein on day 14, 28 and serum was collected by centrifugation for detection of antibody. The level of IgG, IgG1and IgG2a were detected using ELISA kit (Chondrex).

Fourteen days after the first immunization (n = 3 animals per group), spleens were collected and homogenized to obtain a single-cell suspension. Splenocytes were incubated with red cell lysis buffer to remove the red cells. For intracellular cytokine staining of CD4^+^ T cells, splenocytes were incubated at 37°C for 6 h with OVA (100 μg/ml) and brefeldin A (5 μg/ml). Then, the cells were stained with APC-conjugated anti-mouse CD4 and PE-conjugated anti-mouse IFN-γ. For intracellular cytokine staining of CD8^+^ T cells, splenocytes were incubated at 37°C for 6 h with SIINFEKL (2 μg/ml) and brefeldin A (5 μg/ml). Then, cells were stained with APC-conjugated anti-mouse CD8a and PE-conjugated anti-mouse IFN-γ. The samples were analyzed using flow cytometry.

### Statistical Analysis

All numerical values are given as average values ± standard deviation. Statistical analysis was performed using Prism Software (GraphPad, United States), followed by one-way ANOVA or *t*-test analysis. *p* < 0.05 were considered statistically significant.

## Results and Discussion

### Characterization of acGM NPs

**FIGURE 2:**
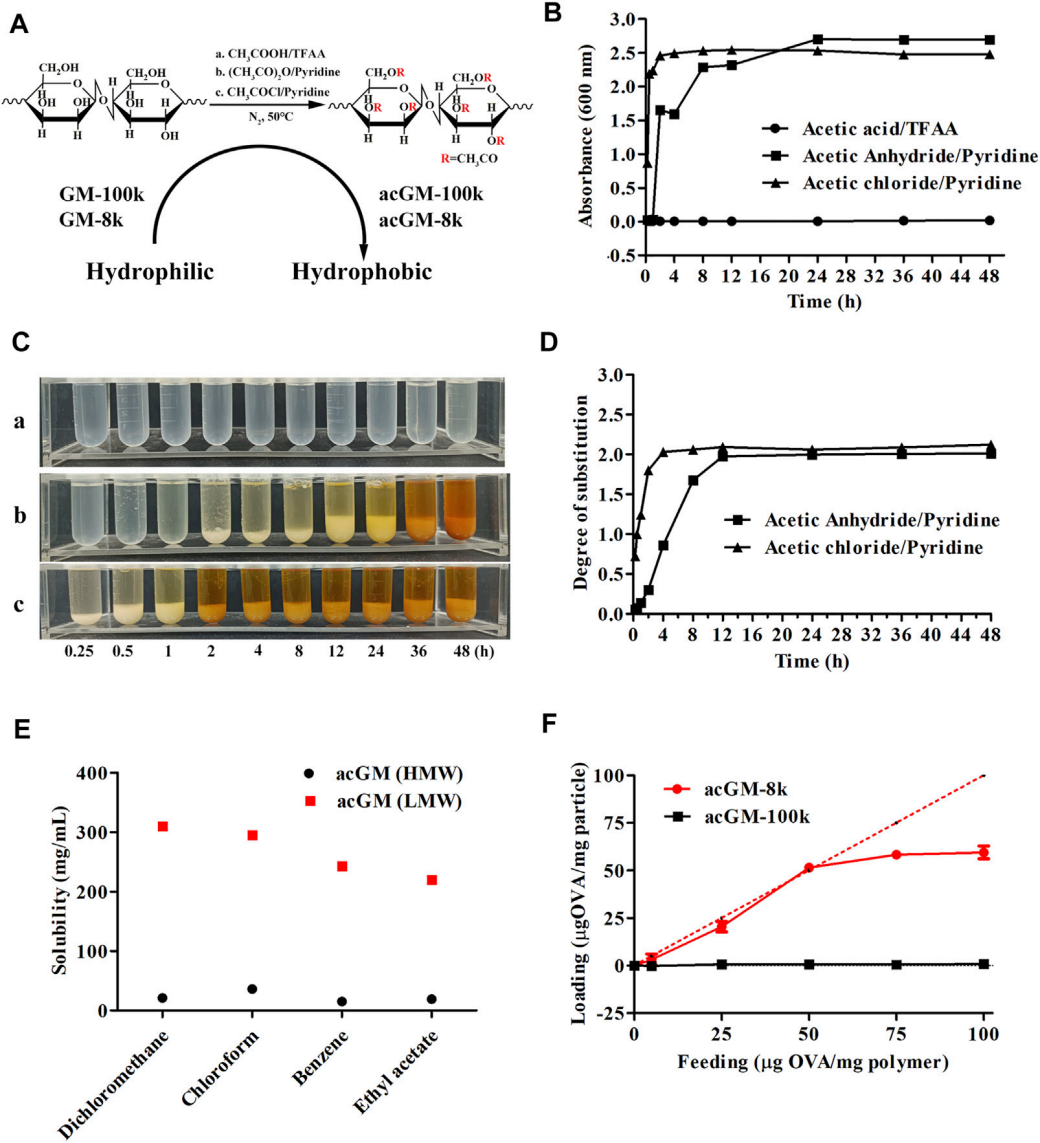
Screening acGM for antigen encapsulation. **(A)** Scheme of different acetylation methods to synthesize acGM. **(B)** Turbidity of the mixture of the reaction solution and dd-H_2_O (V/V = 1:1) at designed time points. **(C)** The mixture of the reaction solution and dd-H_2_O (V/V = 1:1) was precipitated overnight. (a) TFAA/acetic acid; (b) Acetic anhydride/pyridine; (c) Acetic chloride/pyridine. **(D)** Effect of reaction time on the measurement of the degree of substitution (DS). **(E)** Effect of molecular weight on the solubility of acGM with the DS of 1.8 in volatile solvents. **(F)** Effect of molecular weight on OVA loading capacity and encapsulation efficiency.

Three methods of acetylation, one catalyzed by TFAA and two by pyridine, were explored to synthesize acGM with a DS of 1.8 (Figure 2A). Considering that molecular weight directly affects reaction efficiency and solubility, GM with different molecular weights (GM-100k: ∼100 kDa, GM-8k: ∼8 kDa) were used as starting materials. TFAA-catalyzed acetylation of GM-100k was conducted as previously reported by our group (Mu et al., 2021). The average molecular weight of acGM-100k with a DS of 1.8 was 68,414 Da (Supplementary Table S1), indicating that GM was probably cleaved during acetylation. Pyridine-catalyzed acetylation of GM-100k yielded products with low degrees of substitution (DS < 0.4, Supplementary Table S2), despite efforts in increasing the temperature and the concentration of the acetylating reagent.

During acetylation of GM-8k, the products were precipitated by dd-H_2_O. We employed a turbidity test and measured the absorbance at 600 nm to monitor the reaction. The rationale is: acetylation increases the hydrophobicity of GM, so the suspended acGM in water scatters and decreases the amount of visible light penetrating the quartz cell, which can be reflected by lower absorbance reading (Voichick et al., 2017). The data are shown in Figures 2B,C. The mixture of TFAA-catalyzed acetylation solution and dd-H_2_O was transparent (Figures 2C–a), and the turbidity value was low (Figure 2B), suggesting that TFAA-catalyzed acetylation did not yield the product. This is probably due to the hydrolysis of the polysaccharide backbone under the strong acid environment. For pyridine-catalyzed acetylation, either acetic anhydride or acetyl chloride was used as the acetylating reagent, acGM-8k could be obtained by precipitation. The increased turbidity of the acGM-8k suspension revealed that water-insoluble acGM-8k was formed (Figure 2B). The only difference is that acetyl chloride showed a faster acetylation rate than acetic anhydride under the same reaction condition. acGM-8k prepared by acetic anhydride and acetyl chloride can be precipitated at 2 and 0.25 h (Figure 2C), respectively. The DS of the acetyl group increased as the reaction proceeded until it reached a plateau (Figure 2D). The acGM-8k used in subsequent experiments was prepared using acetic anhydride, because the reaction rate of this reagent is moderate and more controllable compared with acetic chloride. The average molecular weight of acGM-8k with a DS of 1.8 was ∼12 kDa, indicating no degradation occurred during acetylation.

acGM-100k and acGM-8k with a DS of 1.8 were used to encapsulate the OVA by double emulsion method (Broaders et al., 2009). Briefly, acGM-100k or acGM-8k was dissolved in a volatile solvent. Then, OVA solution was added, and the mixture was sonicated to obtain a W/O emulsion. The W/O was then added to the 3% PVA solution and the mixture was sonicated to obtain W/O/W emulsion. The W/O/W emulsion was stirred at room temperature to evaporate the volatile solvent. To encapsulate OVA, the hydrophobic acGM needs to be dissolved in a volatile solvent. Therefore, the solubility of acGM-100k and acGM-8k in commonly used volatile solvents was investigated. As shown in Figure 2E, compared to the acGM-100k, acGM-8k with the same DS (1.8) had higher solubility in volatile solvents such as dichloromethane, chloroform, benzene and ethyl acetate. For acGM-100k, the highest solubility was achieved in chloroform (35.6 mg/ml). While the solubility of acGM-8k achieved a high solubility of 310 mg/ml in dichloromethane. Then the acGM-100k and acGM-8k were dissolved in chloroform and dichloromethane to encapsulate OVA, respectively. As shown in Figure 2F, the encapsulation efficiency is very low at feeding values ranging from 5 to 100 μg of protein per mg of acGM-100k. This is mainly due to the poor solubility of acGM-100k in the volatile solvent. For acGM-8k, the loading capacity was found to be nearly quantitative at feed value ranging from 5 to 50 ug protein per mg acGM-8k, which was beneficial for the encapsulation of proteins that are difficult to obtain in large quantities. The encapsulation efficiency decreased to some extent at higher feed values. For subsequent experiments, the feeding value of 50 ug protein per mg acGM-8k was used.

**FIGURE 3:**
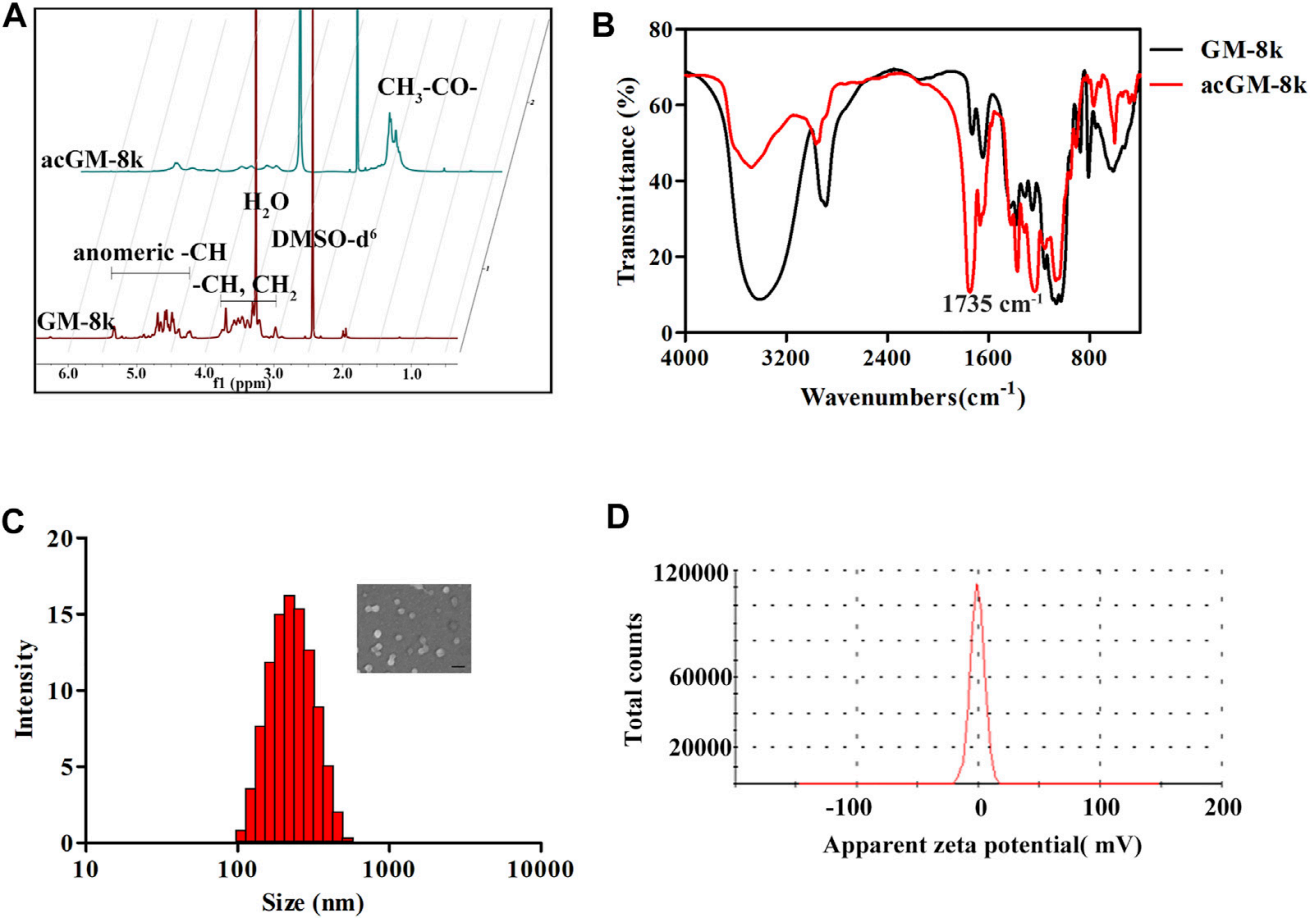
Characterization of acGM-8k and acGM-8k nanoparticles (NPs). **(A)** 600 MHz 1D ^1^H-NMR spectrum of GM-8k and acGM-8k. **(B)** The infrared spectra of GM-8k and acGM-8k. **(C)** Particle size distribution and morphology of acGM-8k NPs (inserted picture, scale bar: 200 nm). **(D)** Zeta potential of acGM-8k NPs.

The acGM-8k was confirmed by ^1^H-NMR spectroscopy and Fourier-transform infrared spectroscopy (FTIR). The peaks in the region of 3.3–5.0 ppm for GM were shifted towards high delta (3.7–5.5 ppm) for acGM-8k due to de-shielding on -CH generated by the acetylation of hydroxyl groups (Figure 3A). The peaks at 2.1 ppm represented the formation of acGM-8k. FTIR results further confirmed the presence of the acetyl group with the appearance of corresponding peaks at ∼1735 cm^−1^ (Figure 3B). The acetyl dextran (acDEX) was synthesized using the same method and the DS of acetyl group was shown in Supplementary Table S3. The nanoparticles were prepared through double-emulsion. The blank nanoparticles, acGM-8k NPs, was prepared without any antigen. As shown in Figure 3C, the acGM-8k NPs were spherical in shape. The zeta potential distribution was displayed in Figure 3D. The average size and zeta potential were 245.7 ± 4.1 nm and -0.216 ± 0.172 mV (Supplementary Table S4), respectively.

### TLR2 Activation by acGM NPs

**FIGURE 4:**
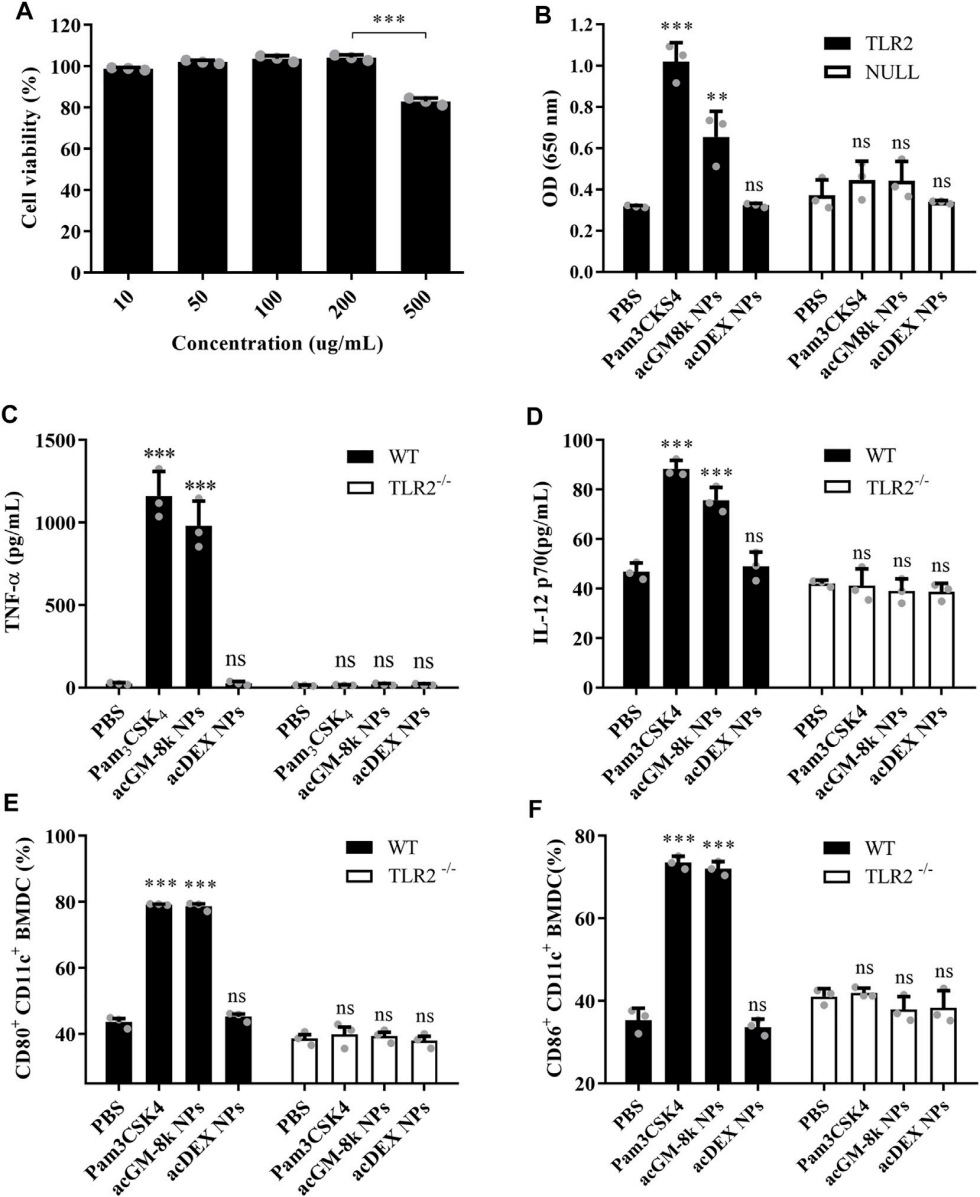
acGM-8k NPs activate BMDCs through TLR2 *in vitro*. **(A)** Cell viability of acGM-8k NPs at different concentrations. **(B)** Detection of secreted embryonic alkaline phosphatase (SEAP) activity in TLR2 reporter cell (murine TLR2-expressing HEK 293 cells, Invivogen). HEK-Blue™ Null2 Cells were used as a negative control. **(C–D)** Determination of cytokines **(C)** TNF-α and **(D)** IL-12p70 secreted by BMDCs isolated from TLR2^−/−^ mice or WT mice. **(E–F)** Percentage of **(E)** CD80^+^ and **(F)** CD86^+^ in CD11c^+^ cells isolated from TLR2^−/−^ mice or WT mice. TLR2^−/−^: TLR2 knockout. WT: wild type. *p < 0.05; **p < 0.01; ***p < 0.001 vs the PBS treatment; ns: no significance; *n* = 3: For **(A)** and **(B)**, data were obtained from three replicates of one representative experiment out of three; for **(C)**–**(F)**, data were obtained from three mice in each group. All numerical values are given as average values ± standard deviation. Statistical analysis was performed using Prism Software (GraphPad, United States), followed by one-way ANOVA analysis with Dunnett’s post hoc evaluation.

The toxicity of acGM-8k NPs was tested in DC2.4 cells. As shown in Figure 4A, the viability of DC2.4 cells remained relatively high after incubation with acGM-8k NPs for 24 h at a concentration range from 10 to 200 μg/ml. Even though a slight decrease at the concentration of 500 μg/ml was observed, the overall viability was above 80%. For subsequent cell experiments, a dose of 100 μg/ml acGM-8k NPs was used.

TLR2 reporter cell line and BMDC isolated from TLR2 knockout mice were used to investigate TLR2 activation. Pam_3_CSK_4_, a TLR2 agonist, was used as a positive control. PBS and acDEX NPs were used as a negative control. As shown in Figure 4B, acGM-8k NPs induced a strong response which was comparable to that of Pam_3_CSK_4_, while acDEX NPs with the same DS (Supplementary Table S3) and size (Supplementary Table S4) did not show any response, suggesting that GM backbone was important for TLR2 activation. This result was consistent with our previous finding (Feng et al., 2019). To further confirm the TLR2 activation of acGM-8k NPs, BMDCs were incubated with acGM-8k NPs and the secreted pro-inflammatory cytokines were determined by ELISA. As shown in Figures 4C,D, acGM-8k NPs significantly increased the secretion of TNF-α (Figure 4C), IL12p70 (Figure 4D), Il-1β (Supplementary Figure S1A) and IFN-γ (Supplementary Figure S1B) in BMDCs isolated from wild type mice compared to PBS group while there is no change in BMDCs isolated from TLR2 knockout mice. This indicates that TLR2 of BMDCs was indeed activated by acGM-8k NPs. The TLR2 activation also increased the expression of CD80/86 (Figures 4E,F; Supplementary Figure S1C,D) and CD40 (Supplementary Figure S1E,F). These data suggest that acGM-8k NPs can be explored as a novel adjuvant candidate in vaccines due to their ability to activate the innate immunity.

### Cellular Uptake of OVA@acGM-8k NPs

**FIGURE 5:**
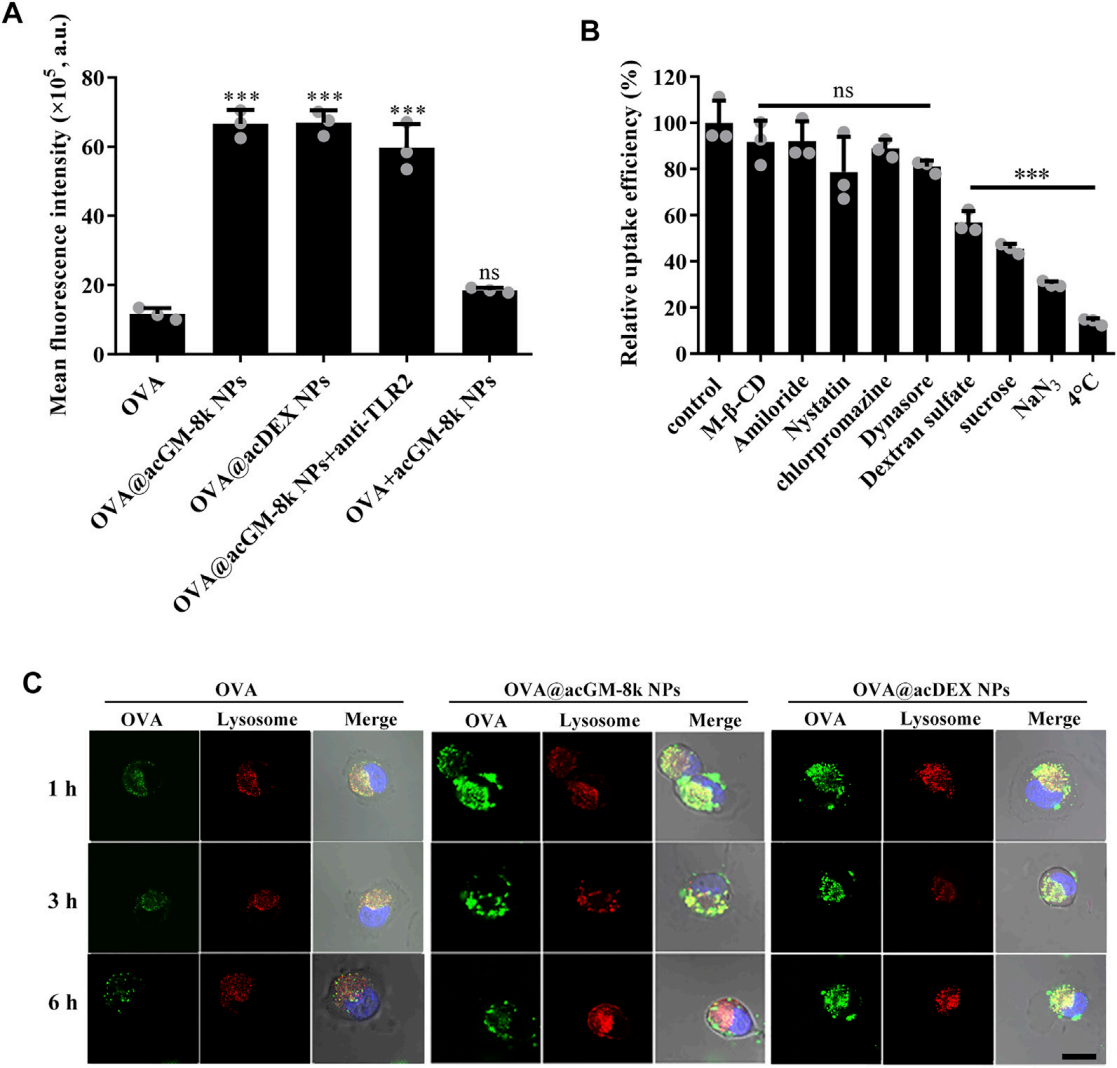
acGM-8k NPs enhanced cellular uptake of OVA. **(A)** Cellular uptake efficiency of OVA@acGM-8k NPs. **(B)** Screening of potential mechanisms of OVA@acGM-8k NPs internalization by DC2.4 cells. **(C)** Confocal microscopy imaging of OVA@acGM-8k NPs in DC2.4 cells (scale bar: 10 μm). Green: FITC-OVA; Red: lysotracker red; Blue: DAPI. ***p < 0.001 vs the control group; ns: no significance; *n* = 3: data were obtained from three replicates of one representative experiment out of three. All numerical values are given as average values ± standard deviation. Statistical analysis was performed using Prism Software (GraphPad, United States), followed by one-way ANOVA analysis with Dunnett’s post hoc evaluation.

Efficiently delivering antigen to the antigen processing cells, namely dendritic cells, is very important for vaccine development. The DC2.4 cells were treated with different formulations for 2 h and the mean fluorescence intensity was determined by flow cytometry. As shown in Figure 5A, OVA@acGM-8k NPs with TLR2 activation activity and OVA@acDEX NPs with no TLR2 activation activity significantly enhanced the cellular uptake of OVA compared with OVA alone. The mean fluorescence intensity of cells treated with OVA@acGM-8k NPs was 5.73 times that treated with OVA. The mixture of OVA and acGM-8k NPs showed no difference in cellular uptake compared with OVA group. These data suggest that the carrier facilitated cellular uptake. It is interesting that the blocking of TLR2 with TLR2 antibody did not influence the cellular uptake of OVA@acGM-8k NPs, which indicates that TLR2 was mainly involved in detecting pathogen-associated molecular patterns rather than internalizing OVA@acGM-8k NPs. To explore the internalization mechanism of OVA@acGM-8k NPs, DC2.4 cells were pretreated with distinct inhibitors of endocytic pathways. As shown in Figure 5B, the uptake efficiency significantly decreased when DC2.4 cells were pretreated with sodium azide (reduced by 70%) or incubated at 4°C (reduced by 87%), suggesting that internalization of OVA@acGM-8k NPs was energy-dependent. Dynasore and chlorpromazine did not inhibit the cellular uptake, suggesting that dynamin and clathrin were not involved in OVA@acGM-8k NPs internalization. In addition, methyl-β-cyclodextrin, nystatin, and amiloride did not significantly affect OVA@acGM-8k NPs uptake, indicating that caveolae/lipid raft-mediated endocytosis or micropinocytosis were not involved. Sucrose significantly inhibited the endocytosis of OVA@acGM-8k NPs, suggesting the involvement of non-selective endocytosis (Guo et al., 2015). Uptake was reduced by about 40% in the presence of dextran sulfate, suggesting that OVA@acGM-8k NPs were internalized mainly through scavenger receptor-mediated endocytosis.

The intracellular trafficking was observed by confocal laser scanning microscopy. As shown in Figure 5C and Supplementary Figure S2, OVA and OVA@acGM-8k NPs and OVA@acDEX NPs were trapped at lysosomes at 1 and 3 h since the green fluorescence was colocalized with the red fluorescence as yellow dots. These results were consistent with the fact that scavenger receptor-mediated endocytosis mainly transfers internalized nanoparticles to lysosome (Canton et al., 2013). In addition, OVA@acGM-8k NPs completely escaped from lysosomes by 6 h. While most of OVA@acDEX NPs were still trapped in lysosome at 6 h, with a small part of OVA@acDEX NPs escaping from lysosomes. The OVA delivered to the cytoplasm by OVA@acGM-8k NPs and OVA@acDEX NPs might also be processed by the proteasome and presented to MHC-I restricted T cells.

### *In vivo* Biodistribution of OVA@acGM-8k NPs and DC Maturation

**FIGURE 6:**
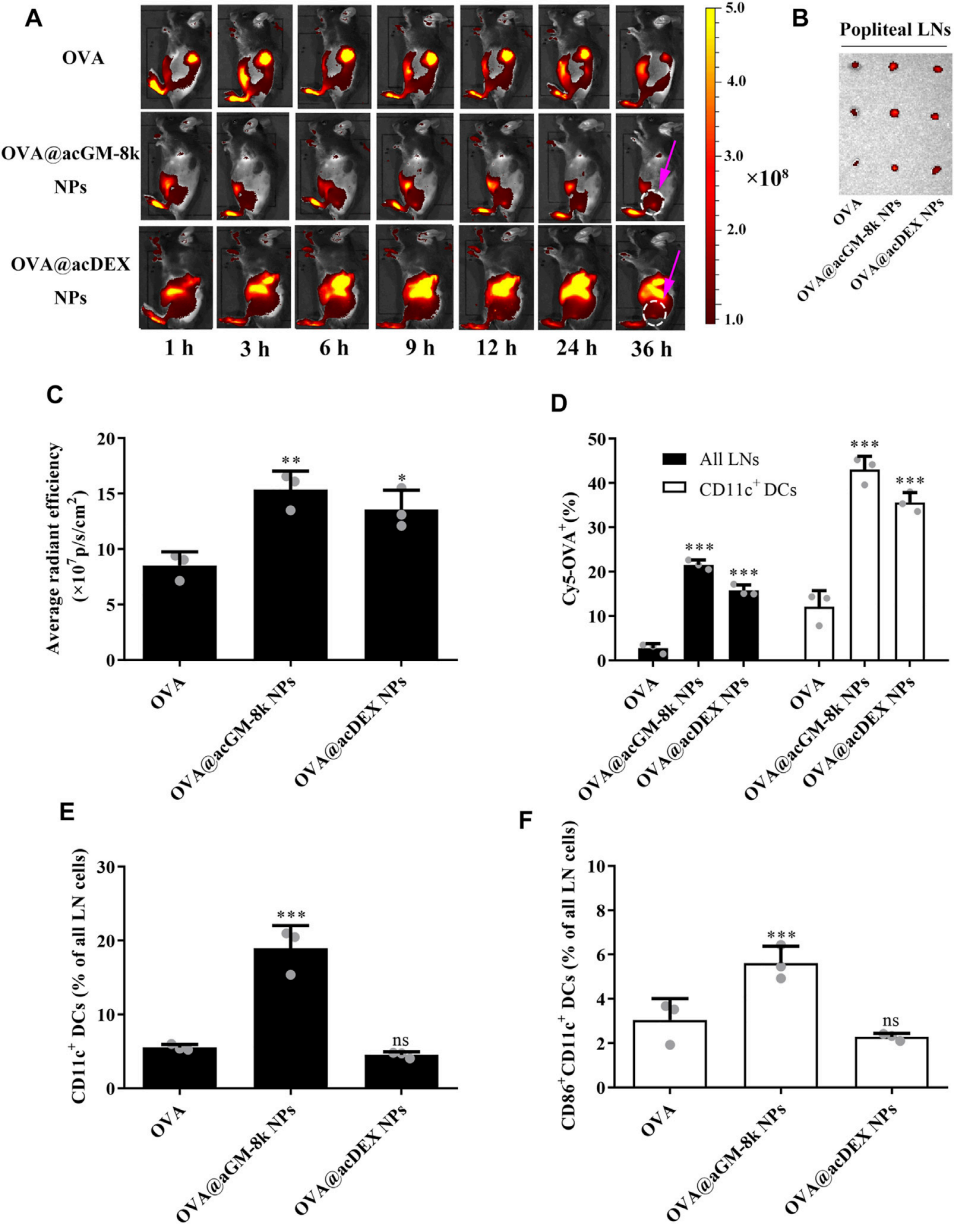
Distribution of OVA@acGM-8k NPs and maturation of lymph node-resident DC *in vivo*. **(A)** Migration of OVA@acGM-8k NPs from the injection site to popliteal lymph nodes (LNs) *in vivo*. The pink arrow indicates the popliteal LNs. **(B)** Popliteal LNs were isolated and visualized at 36 h after footpad injection. **(C)** Average radiant efficiency of popliteal LNs at 36 h after footpad injection. **(D)** The percentages of Cy5-positive in all LNs and CD11c^+^ DCs 36 h after footpad injection. **(E)** The percentage CD11c^+^ DCs in LNs 72 h after immunization. **(F)** The percentage of CD86^+^ in CD11c^+^ DCs in LNs 72 h after immunization. *p < 0.05, **p < 0.01; ***p < 0.001 vs the OVA group; ns: no significance; *n* = 3: For **(B)**–**(F)**, data were obtained from three mice in each group. All numerical values are given as average values ± standard deviation. Statistical analysis was performed using Prism Software (GraphPad, United States), followed by one-way ANOVA analysis with Dunnett’s post hoc evaluation.

The biodistribution of OVA@acGM-8k NPs was investigated as an adaptive response initiated in lymph nodes (LNs). All formulations for imaging contained OVA labelled with Cyanine 5 (Cy5). Briefly, free OVA, OVA@acGM-8k NPs and OVA@acDEX NPs were subcutaneously injected into the footpad of mice. At designed time points, mice were visualized by IVIS Spectrum system. As shown in Figure 6A, significant fluorescence signals of Cy5 from OVA@acGM-8k NPs and OVA@acDEX NPs were detected in popliteal LNs at 6 h (Supplementary Figure S3) and 9 h, respectively. The signal continued to increase until 36 h, while that from free OVA was relatively low. After 36 h, popliteal LNs were harvested and visualized. The fluorescence intensity in popliteal LNs was semi-quantified. The *ex vivo* images (Figure 6B) and semi-quantitative results (Figure 6C) showed that both OVA@acGM-8k NPs and OVA@acDEX NPs accumulated to a much greater extent than free OVA in popliteal LNs, suggesting that nanoparticles could enhance the accumulation of OVA in popliteal LNs (Figure 6C). Notably, strong signals also appeared in other organs such as the liver and gallbladder for OVA@acDEX NPs and free OVA treated mice. Such a phenomenon was not observed in OVA@acGM-8k NPs. These results indicated that OVA@acGM-8k NPs could specifically accumulate in LNs.

Flow cytometry was used to explore the internalization of OVA@acGM-8k NPs by dendritic cells *in vivo*. As shown in Figure 6D and Supplementary Figure S4, both OVA@acGM-8k NPs and OVA@acDEX NPs increased the uptake by lymph node cells compared with free OVA. About 21 and 15% LNs cells were Cy5-positive at 36 h after injection with OVA@acGM-8k NPs and OVA@acDEX NPs, respectively. While only 2.7% of lymph node cells were Cy5-positive. 43% of CD11c^+^ DCs in LNs were Cy5-positive after treatment with OVA@acGM-8k NPs, which was significantly higher than that of OVA@acDEX NPs (35%) and free OVA (12%). Together, these results showed that OVA@acGM-8k NPs efficiently delivered OVA to lymph node cells, especially CD11c^+^ DCs.

Dendritic cells must be activated to express co-stimulatory molecules to achieve efficient antigen presentation. Therefore, the potential of OVA@acGM-8k NPs to expand and activate DCs in LNs was investigated. A greater proportion of DCs in LNs was detected in animals injected with OVA@acGM-8k NPs (18.9%) than that injected with OVA@acDEX NPs (4.53%) and free OVA (5.54%) (Figure 6E, Supplementary Figure S5A). OVA@acGM-8k NPs led to higher expression of co-stimulatory CD86 by DCs in LNs than OVA@acDEX NPs and free OVA (Figure 6F, Supplementary Figure S5B). These results suggest that OVA@acGM-8k can activate the dendritic cells *in vivo*.

### Activation of Humoral Immune Responses by OVA@acGM-8k NPs

**FIGURE 7:**
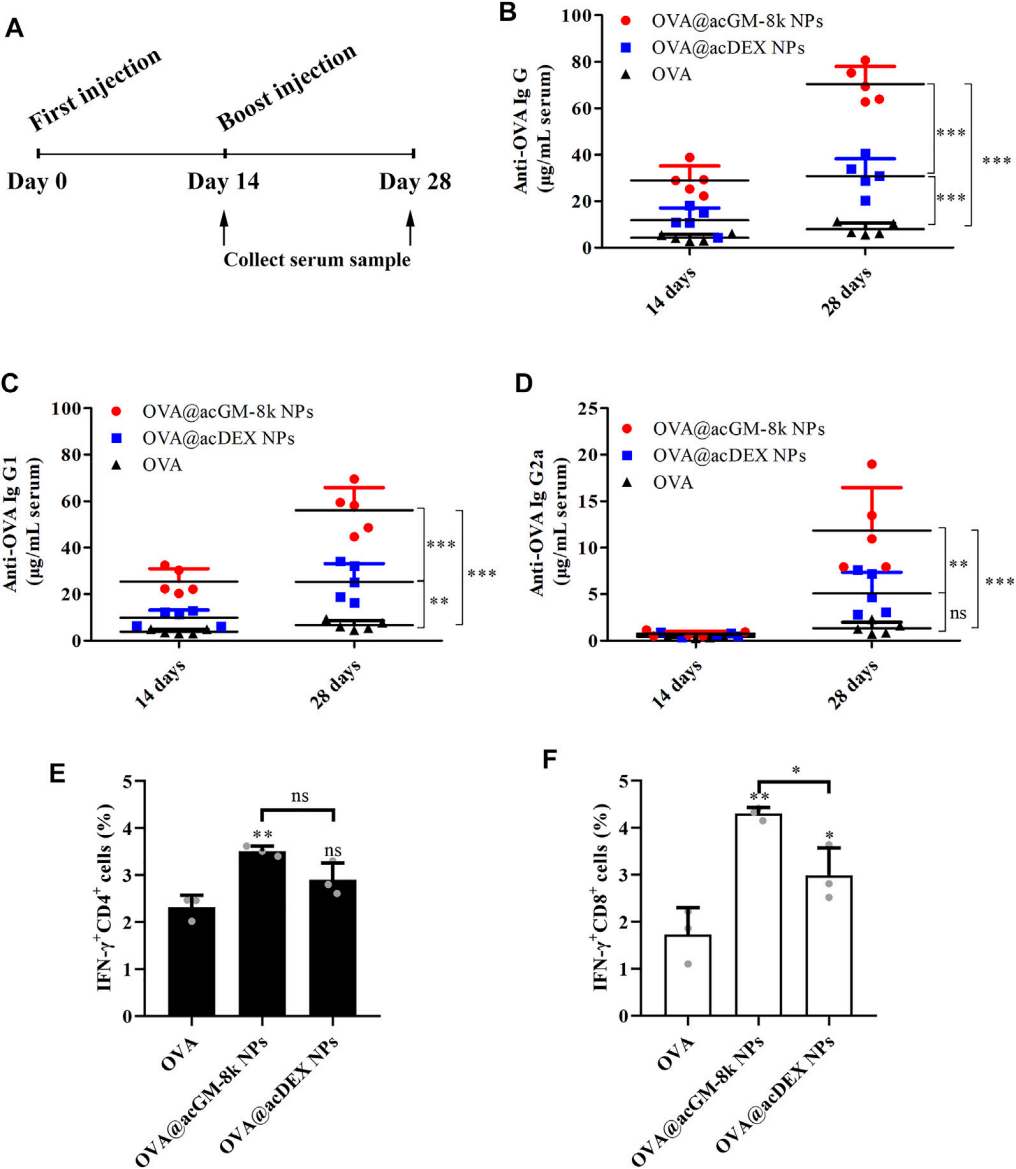
OVA@acGM-8k NPs enhanced the humoral and cellular immune response. **(A)** Schematic of the experiment design. **(B–D)** Levels of anti-OVA IgG, IgG1, and IgG2a antibodies in serum were determined. **(E–F)** Splenocytes were restimulated with OVA (100 μg/ml) or SIINFEKL (2 μg/ml) for 6 h at 37°C; then, flow cytometry was used to determine percentages of OVA-specific **(E)** IFN-γ–producing CD4^+^ T cells and **(F)** IFN-γ-producing CD8^+^ T cells. *p < 0.05, **p < 0.01, ***p < 0.001 vs the OVA group; ns: no significance. For **(B)**–**(D)**, data were obtained from five mice in each group; For **(E)**–**(F)**, data were obtained from three mice in each group. All numerical values are given as average values ± standard deviation. Statistical analysis was performed using Prism Software (GraphPad, United States), followed by one-way ANOVA analysis with Dunnett’s post hoc evaluation.

On day 0 and 14, mice were subcutaneously injected with 10 μg OVA or 200 μg OVA@acGM-8k NPs or 200 μg OVA@acDEX NPs containing 10 μg OVA. The serum was collected on day 14 and day 28 (Figure 7A). The IgG, IgG1 and IgG2a levels were determined to characterize humoral immunity (Hong et al., 2020). As shown in Figures 7B–D, both OVA@acGM-8k NPs and OVA@acDEX NPs induced significantly higher antibody responses than free OVA, with one exception that OVA@acDEX NPs did not increase IgG2a titers compared to free OVA (Figure 7D). The antibodies (IgG, IgG1 and IgG2a) induced by OVA@acGM-8k NPs were significantly higher than that by OVA@acDEX NPs. These data suggest that acGM-8k NPs can be explored as a novel adjuvant candidate in vaccines due to its ability to link the innate with the adaptive immune response.

Our data suggest that OVA@acGM-8k NPs can boost the humoral immune response with the help of carrier and TLR2 activation. Commonly, total IgG is related to general humoral immune response and a high IgG2a/IgG1 ratio is related to Th1-type immune response (Gamboa-Leon et al., 2014). Fourteen days after the first immunization, the ratio of IgG2a/IgG1 of OVA@acGM-8k treated mice was maintained at a low level (2.94%), indicating that Th2-type immune response was dominant in the early stage of immunization. Twenty-eight days after the first immunization, the ratio of IgG2a/IgG1 increased to 21.12%, suggesting that Th1-type immune response increased, which was crucial for clearing the intracellular pathogens (Rostamian et al., 2017). Although this ratio is also increasing for OVA and OVA@acDEX NPs treated mice, this ratio is not statistically significant when characterizing the type of immune effect (Th1 or Th2) due to low antibody titers.

To investigate the T cell response, splenocytes of vaccinated mice were harvested on day 14 for *ex vivo* culture and restimulation with OVA or SIINFEKL (OVA-specific, MHC-Ⅰ-restricted peptide). Brefeldin A was added to inhibit protein secretion via specific pathways of intracellular vesicular trafficking. The proportions of CD4^+^ and CD8^+^ T cells positive for interferon-γ (IFN-γ) were determined using flow cytometry. OVA@acGM-8k NPs significantly increased the production of IFN-γ by both CD4^+^ (Figure 7E) and CD8^+^ cells (Figure 7F). These experiments revealed that OVA@acGM-8k NPs could activate an effector phenotype in CD4^+^ and CD8^+^ T cells. It is interesting that OVA@acDEX NPs without the effect of DCs’ maturation could also induce CD8^+^ T cell response (Figure 7F), probably due to the escape of OVA@acDEX NPs from lysosomes (Shen et al., 2006). The acDEX NPs enhanced substantial cellular uptake of OVA and promoted the escape of OVA to the cytoplasm, where antigen is processed by the proteasome and presented to CD8^+^ T cells.

### *In vivo* Safety of OVA@acGM-8k NPs

**FIGURE 8:**
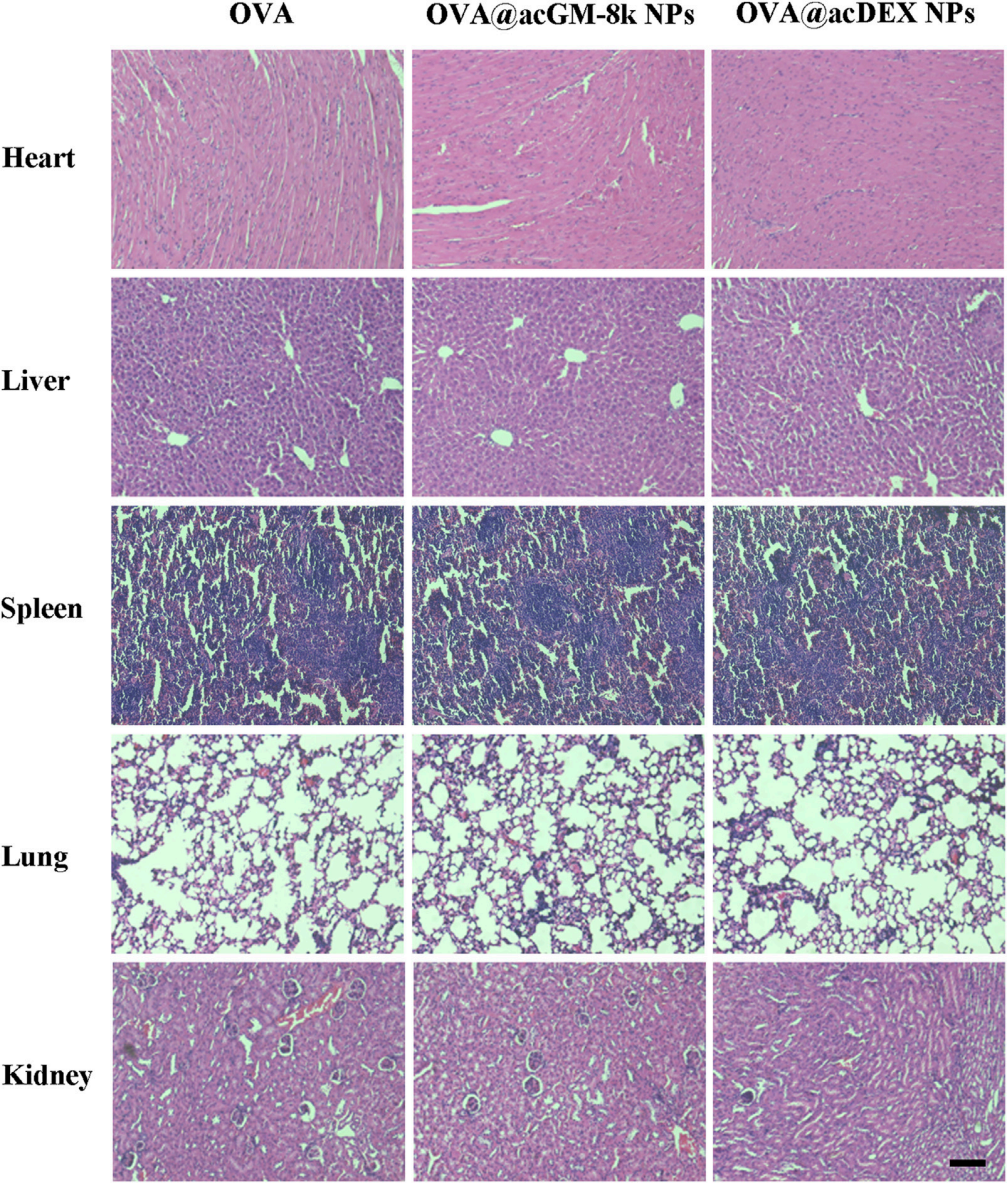
Systemic toxicities of OVA@acGM-8k NPs. Histological evaluation of heart, liver, spleen, lung, and kidney. Mice were subcutaneously injected with OVA, OVA@acGM-8k NPs and OVA@acDEX NPs at an interval of 2 weeks. 14 days after the last injection, mice were sacrificed and the heart, liver, spleen, lung, and kidney were separated for paraffin section and H&E staining. Scale bar: 100 μm.

Systemic toxicity of OVA@acGM-8k NPs was investigated. Fourteen days after the last immunization, the heart, lung, liver, spleen, and kidney of immunized mice were dissected for hematoxylin and eosin staining (Figure 8). No myocardium and epicardial abnormalities were observed in the heart tissue for all groups. The ratio of spleen red pulp and white pulp were within the normal range. The number and volume of the kidney glomeruli and renal tubular epithelium were also normal in all groups. The lung and liver tissues of the OVA@acGM-8k NPs and OVA@acDEX NPs immunized mice and the free OVA immunized mice were normal.

Adjuvants are also related to their adverse effects, including acute and long-term ones. Our design showed high safety in mice, with no acute toxicity or abnormal responses observed throughout the test period. The efficient accumulation of OVA@acGM-8k NPs in LNs enables clearer understanding and prediction of the *in vivo* distribution, which is positive for assessing the safety of this system. One fundamental advantage of acGM is its relatively simple structure–a mannose-glucose polysaccharide backbone modified with acetyl groups–which excludes lipids or metals that as adjuvant ingredients might bring about side effects to the human body. It does not contain microbe-derived components, either. Although long-term experiments are demanded in future for a comprehensive evaluation of the *in vivo* fate of this material, the biodegraded products of acGM are largely predictable.

## Conclusion

This study demonstrated the design and evaluation of a self-adjuvant glycan carrier for antigen delivery. It has two main advantages: a desirable antigen loading capacity and TLR-mediated activation of the innate response, leading to efficient humoral and cellular immune responses. acGM, the core material of this system, is prepared via a one-step reaction based on a degradable polysaccharide with good biocompatibility and clear immune activities. It integrates the two roles–a vehicle for delivery and an adjuvant for boosting–in one material, simplifying the procedures and increasing the controllability for vaccine preparation. In mice, the acGM-8k nanocarriers delivering model antigens achieved a favorable immunization efficacy and showed no adverse effects such as toxicity. Future work will focus on long-term evaluation of the safety of this material as well as its applications in specific disease models, such as tumors and infectious diseases, to investigate its therapeutic potential in eliciting corresponding immune responses.

## Data Availability

The original contributions presented in the study are included in the article/Supplementary Material, further inquiries can be directed to the corresponding authors.

## References

[B1] Alving C. R. Peachman K. K. Rao M. Reed S. G. (2012). Adjuvants for Human Vaccines. Curr. Opin. Immunol. 24 (3), 310–315. 10.1016/j.coi.2012.03.008 22521140 PMC3383374

[B2] Apostólico J. d. S. Lunardelli V. A. S. Coirada F. C. Boscardin S. B. Rosa D. S. (2016). Adjuvants: Classification, Modus Operandi, and Licensing. J. Immunol. Res. 2016, 1–16. 10.1155/2016/1459394 PMC4870346 27274998

[B3] Broaders K. E. Cohen J. A. Beaudette T. T. Bachelder E. M. Fréchet J. M. J. (2009). Acetalated Dextran Is a Chemically and Biologically Tunable Material for Particulate Immunotherapy. Proc. Natl. Acad. Sci. U.S.A. 106 (14), 5497–5502. 10.1073/pnas.0901592106 19321415 PMC2666992

[B4] Canton J. Neculai D. Grinstein S. (2013). Scavenger Receptors in Homeostasis and Immunity. Nat. Rev. Immunol. 13 (9), 621–634. 10.1038/nri3515 23928573

[B5] Feng Y. Mu R. Wang Z. Xing P. Zhang J. Dong L. (2019). A Toll-like Receptor Agonist Mimicking Microbial Signal to Generate Tumor-Suppressive Macrophages. Nat. Commun. 10 (1), 2272. 10.1038/s41467-019-10354-2 31118418 PMC6531447

[B6] Gamboa-Leon R. Vera-Ku M. Peraza-Sanchez S. R. Ku-Chulim C. Horta-Baas A. Rosado-Vallado M. (2014). Antileishmanial Activity of a Mixture ofTridax procumbensandAllium Sativumin Mice. Parasite 21, 15. 10.1051/parasite/2014016 24717526 PMC3980668

[B7] Garçon N. Di Pasquale A. (2017). From Discovery to Licensure, the Adjuvant System story. Hum. Vaccin. Immunother. 13 (1), 19–33. 27636098 10.1080/21645515.2016.1225635 PMC5287309

[B8] Garçon N. Tavares Da Silva F. Schijns V. E. J. C. O'Hagan D. T. (2017). “Chapter 15 - Development and Evaluation of AS04, a Novel and Improved Adjuvant System Containing 3-O-Desacyl-4′- Monophosphoryl Lipid A and Aluminum Salt,” in Immunopotentiators in Modern Vaccines. Second Edition (Academic Press), 287–309.

[B9] Guo S. Zhang X. Zheng M. Zhang X. Min C. Wang Z. (2015). Selectivity of Commonly Used Inhibitors of Clathrin-Mediated and Caveolae-dependent Endocytosis of G Protein-Coupled Receptors. Biochim. Biophys. Acta (Bba) - Biomembranes 1848 (10), 2101–2110. Part A. 10.1016/j.bbamem.2015.05.024 26055893

[B10] Hong X. Zhong X. Du G. Hou Y. Zhang Y. Zhang Z. (2020). The Pore Size of Mesoporous Silica Nanoparticles Regulates Their Antigen Delivery Efficiency. Sci. Adv. 6 (25), eaaz4462. 10.1126/sciadv.aaz4462 32596445 PMC7304990

[B11] Lambrecht B. N. Kool M. Willart M. A. Hammad H. (2009). Mechanism of Action of Clinically Approved Adjuvants. Curr. Opin. Immunol. 21 (1), 23–29. 10.1016/j.coi.2009.01.004 19246182

[B12] Martínez A. Bono C. Gozalbo D. Goodridge H. S. Gil M. L. Yáñez A. (2020). TLR2 and Dectin-1 Signaling in Mouse Hematopoietic Stem and Progenitor Cells Impacts the Ability of the Antigen Presenting Cells They Produce to Activate CD4 T Cells. Cells 9 (5), 1317. 10.3390/cells9051317 PMC7290964 32466296

[B13] Mata-Haro V. Cekic C. Martin M. Chilton P. M. Casella C. R. Mitchell T. C. (2007). The Vaccine Adjuvant Monophosphoryl Lipid A as a TRIF-Biased Agonist of TLR4. Science 316 (5831), 1628–1632. 10.1126/science.1138963 17569868

[B14] Mishra S. Upadhaya K. Mishra K. B. Shukla A. K. Tripathi R. P. Tiwari V. K. (2016). Carbohydrate-Based Therapeutics. Stud. Nat. Prod. Chem. 49, 307–361. 10.1016/b978-0-444-63601-0.00010-7

[B15] Mitchell M. J. Billingsley M. M. Haley R. M. Wechsler M. E. Peppas N. A. Langer R. (2021). Engineering Precision Nanoparticles for Drug Delivery. Nat. Rev. Drug Discov. 20 (2), 101–124. 10.1038/s41573-020-0090-8 33277608 PMC7717100

[B16] Mu R. Zhang Y. Yan L. Liao Z. Yang Y. Su H. (2021). A "Bridge‐Building" Glycan Scaffold Mimicking Microbial Invasion for *In Situ* Endothelialization. Adv. Mater. 33 (42), 2103490. 10.1002/adma.202103490 34476850

[B17] Niu Y. Li Q. Xie R. Liu S. Wang R. Xing P. (2017). Modulating the Phenotype of Host Macrophages to Enhance Osteogenesis in MSC-Laden Hydrogels: Design of a Glucomannan Coating Material. Biomaterials 139, 39–55. 10.1016/j.biomaterials.2017.05.042 28582717

[B18] Pati R. Shevtsov M. Sonawane A. (2018). Nanoparticle Vaccines against Infectious Diseases. Front. Immunol. 9 (2224), 2224. 10.3389/fimmu.2018.02224 30337923 PMC6180194

[B19] Piao W. Ru L. W. Toshchakov V. Y. (2016). Differential Adapter Recruitment by TLR2 Co-receptors. Pathog. Dis. 74 (5). 10.1093/femspd/ftw043 PMC5985480 27150837

[B20] Rogovina S. Aleksanyan K. Vladimirov L. Prut E. Ivanushkina N. Berlin A. (2018). Development of Novel Biodegradable Polysaccharide-Based Composites and Investigation of Their Structure and Properties. J. Polym. Environ. 26 (4), 1727–1736. 10.1007/s10924-017-1069-3

[B21] Rostamian M. Sohrabi S. Kavosifard H. Niknam H. M. (2017). Lower Levels of IgG1 in Comparison with IgG2a Are Associated with Protective Immunity against Leishmania Tropica Infection in BALB/c Mice. J. Microbiol. Immunol. Infect. 50 (2), 160–166. 10.1016/j.jmii.2015.05.007 26066544

[B22] Saso A. Kampmann B. (2017). Vaccine Responses in Newborns. Semin. Immunopathol 39 (6), 627–642. 10.1007/s00281-017-0654-9 29124321 PMC5711983

[B23] Shen H. Ackerman A. L. Cody V. Giodini A. Hinson E. R. Cresswell P. (2006). Enhanced and Prolonged Cross-Presentation Following Endosomal Escape of Exogenous Antigens Encapsulated in Biodegradable Nanoparticles. Immunology 117 (1), 78–88. 10.1111/j.1365-2567.2005.02268.x 16423043 PMC1782199

[B24] Sivakumar S. M. Safhi M. M. Kannadasan M. Sukumaran N. (2011). Vaccine Adjuvants - Current Status and Prospects on Controlled Release Adjuvancity. Saudi Pharm. J. 19 (4), 197–206. 10.1016/j.jsps.2011.06.003 23960760 PMC3744968

[B25] Sun X. Stefanetti G. Berti F. Kasper D. L. (2019). Polysaccharide Structure Dictates Mechanism of Adaptive Immune Response to Glycoconjugate Vaccines. Proc. Natl. Acad. Sci. U.S.A. 116 (1), 193–198. 10.1073/pnas.1816401115 30510007 PMC6320544

[B26] Tregoning J. S. Flight K. E. Higham S. L. Wang Z. Pierce B. F. (2021). Progress of the COVID-19 Vaccine Effort: Viruses, Vaccines and Variants versus Efficacy, Effectiveness and Escape. Nat. Rev. Immunol. 21 (10), 626–636. 10.1038/s41577-021-00592-1 34373623 PMC8351583

[B27] Underhill D. M. (2003). Macrophage Recognition of Zymosan Particles. J. Endotoxin Res. 9 (3), 176–180. 10.1177/09680519030090030601 12831459

[B28] van Doorn E. Liu H. Huckriede A. Hak E. (2016). Safety and Tolerability Evaluation of the Use of Montanide ISA51 as Vaccine Adjuvant: A Systematic Review. Hum. Vaccin. Immunother. 12 (1), 159–169. 10.1080/21645515.2015.1071455 26378866 PMC4962750

[B29] Voichick N. Topping D. Griffiths R. (2017). “Technical Note: False Low Turbidity Readings during High Suspendedsediment Concentrations,” in Hydrology and Earth System Sciences Discussions, 1–11.

[B30] Wang Z.-B. Xu J. (2020). Better Adjuvants for Better Vaccines: Progress in Adjuvant Delivery Systems, Modifications, and Adjuvant-Antigen Codelivery. Vaccines 8 (1), 128. 10.3390/vaccines8010128 PMC7157724 32183209

