# Supplementary material for: A Toll-like Receptor-Activating, Self-Adjuvant Glycan Nanocarrier
Source: Front Chem. 2022 May 3;10:864206. doi: 10.3389/fchem.2022.864206 (PMC9110926; doi:10.3389/fchem.2022.864206)
Supplement: Supplementary file 1 [file DataSheet1.docx]

**A toll-like receptor-activating, self-adjuvant glycan nanocarrier**

Daping Xie^1^, Yiming Niu^1^, Ruoyu Mu^1^, Senio Campos de Souza^1^, Xiaoyu Yin^1,2^,

Lei Dong^2,*^, Chunming Wang^1,*^

^1^State Key Laboratory of Quality Research in Chinese Medicine, Institute of Chinese Medical Sciences, University of Macau, Taipa, Macau SAR, China.

^2^State Key Laboratory of Pharmaceutical Biotechnology, School of Life Sciences, Nanjing University, Nanjing 210093, China.

*** Correspondence:**Chunming Wang: [cmwang@umac.mo](mailto:cmwang@umac.mo) & Lei Dong: [leidong@nju.edu.cn](mailto:leidong@nju.edu.cn)

**Supporting information**

Table S1. Mw of GM and acGM

| **Polysaccharide** | **Mw (Da)** |
| --- | --- |
| **GM-100k** | **125787** |
| **GM-8k** | **8200** |
| **acGM-100k** | **68414** |
| **acGM-8k** | **12452** |

**Table S2.** Degree of substitution of acGM-100k prepared by acetic anhydride/pyridine system

| **Parameters** | **DS** |
| --- | --- |
| **50℃，3 equivalent** | 0.30±0.01 |
| **50℃，9 equivalent** | 0.31±0.01 |
| **90℃，3 equivalent** | 0.34±0.02 |
| **90℃，9 equivalent** | 0.36±0.00 |

**Table S3.** Degree of substitution of acetyl group in acGM-8k and acDEX for encapsulation of OVA

| **Acetyl polysaccharide** | **Degree of substitution** |
| --- | --- |
| **acGM-8k** | 1.89±0.17 |
| **acDEX** | 1.81±0.05 |

**Table S4.** Size and zeta potential of blank nanoparticles or nanoparticles loaded with OVA.

| **Nanoparticles** | **Size (nm)** | **Zeta potential (mV)** |
| --- | --- | --- |
| **acGM-8k NPs** | 245.7±4.1 | -0.216±0.172 |
| **acDEX NPs** | 224.4±2.4 | -0.140±0.212 |
| **OVA@acGM-8k NPs** | 231.9±3.5 | -0.087±0.092 |
| **OVA@acDEX NPs** | 229.7±3.7 | -0.225±0.098 |


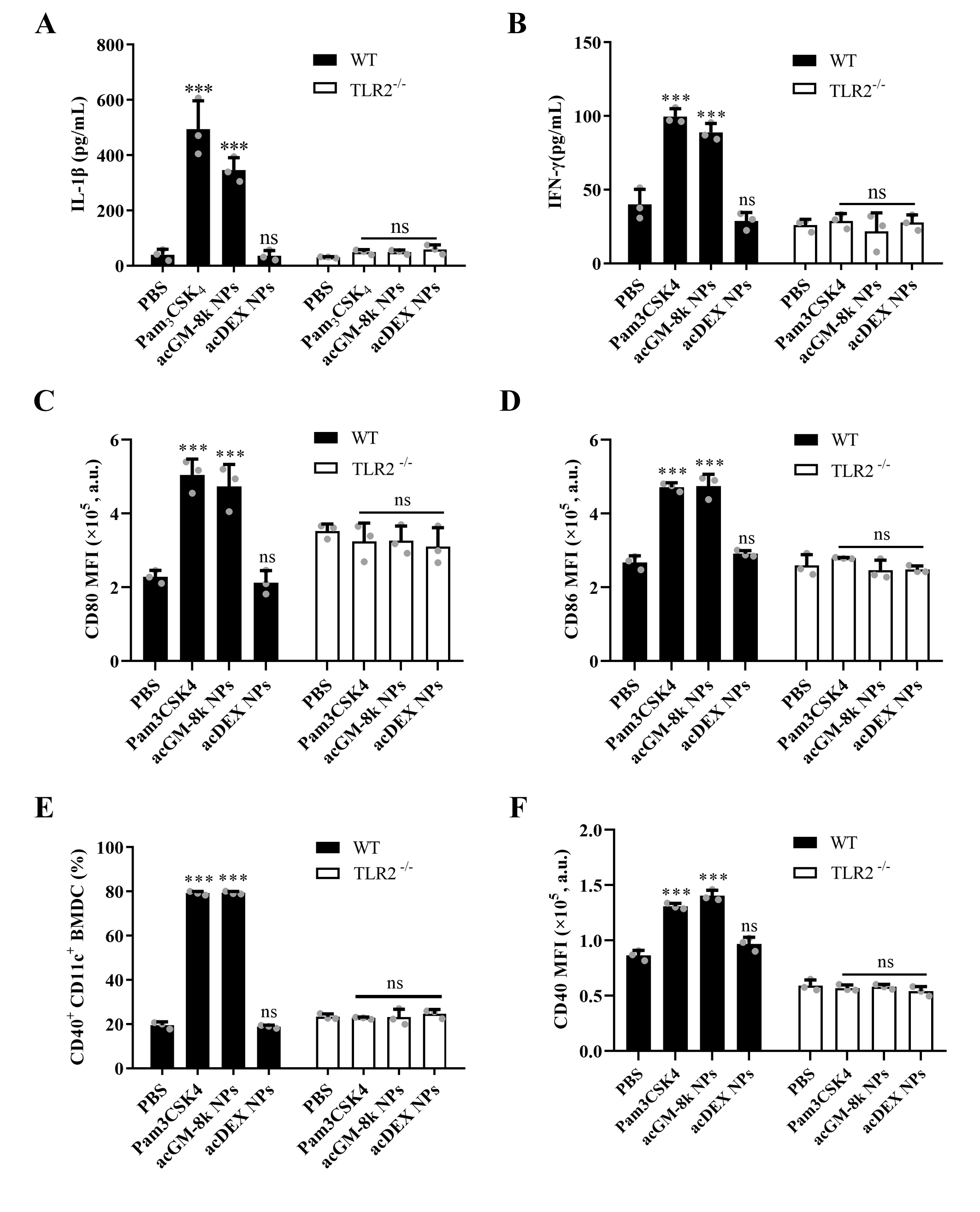


**Figure S1.** acGM-8k NPs activate the BMDCs through TLR2 *in vitro*. (A-B) Determination of cytokines (A) IL-1β and (B) IFN-γ secreted by BMDCs isolated from TLR2^-/-^ mice or WT mice. (C-D). Median fluorescence intensity (MFI) of (C) CD80 and (D) CD86 in CD11c^+^ cells isolated from TLR2^-/-^ mice or WT mice. (E) Percentage CD40^+^ cells in CD11c^+^ cells TLR2^-/-^ mice or WT mice. (F) MFI of CD40 in CD11c^+^ cells isolated from TLR2^-/-^ mice or WT mice. TLR2-/-: TLR2 knockout. WT: wild type. **P* < 0.05, ***P* < 0.01; ****P* < 0.001 vs. the PBS group; ns: no significance; n=3: data was obtained from three mice in each group. All numerical values are given as average values ± standard deviation. Statistical analysis was performed using Prism Software (GraphPad, USA), followed by one-way ANOVA analysis with Dunnett's post hoc evaluation.


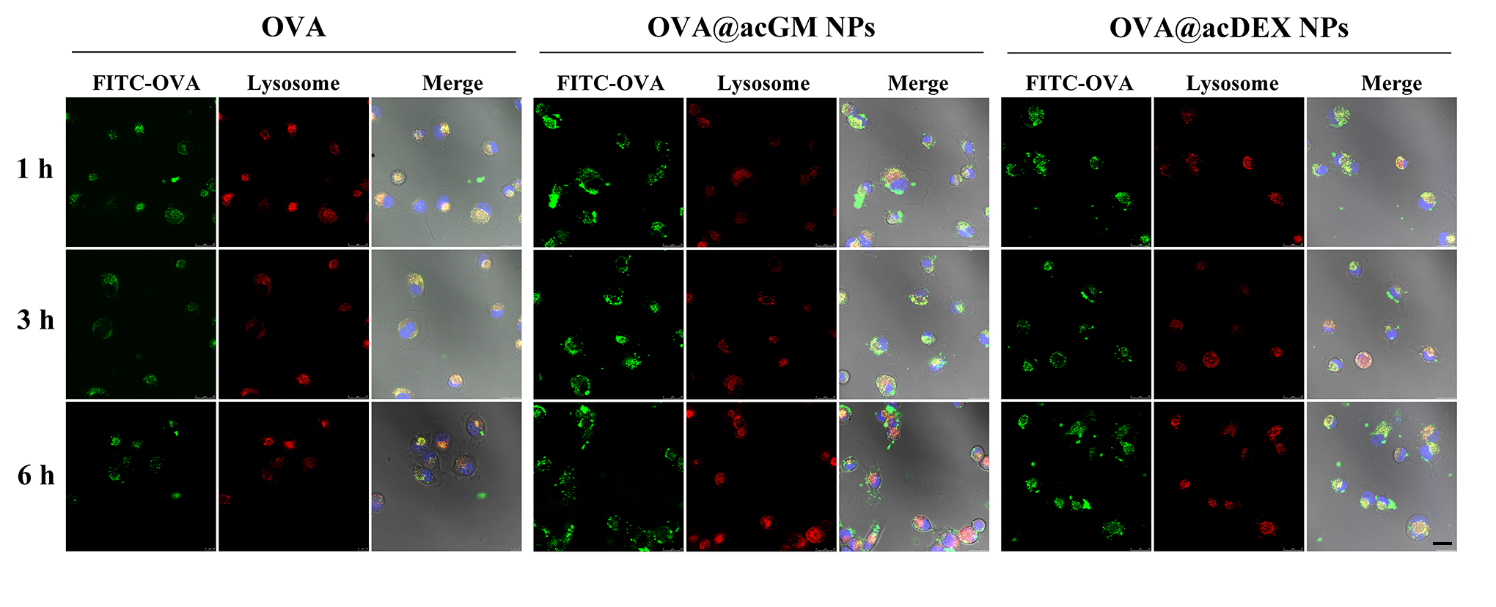


**Figure S2.** Confocal microscopy imaging of OVA@acGM-8k NPs in DC2.4 cells (scale bar: 10 μm). Green: FITC-OVA; Red: lysotracker red; Blue: DAPI.





**Figure S3.** Average radiant efficiency of popliteal LNs 3 and 6 h after footpad injection, respectively. **P* < 0.05, ***P* < 0.01; ****P* < 0.001 vs. the 3 h; ns: no significance; *n* = 3: data were obtained from three mice in each group. All numerical values are given as average values ± standard deviation. Statistical analysis was performed using Prism Software (GraphPad, USA), followed by t-test analysis.


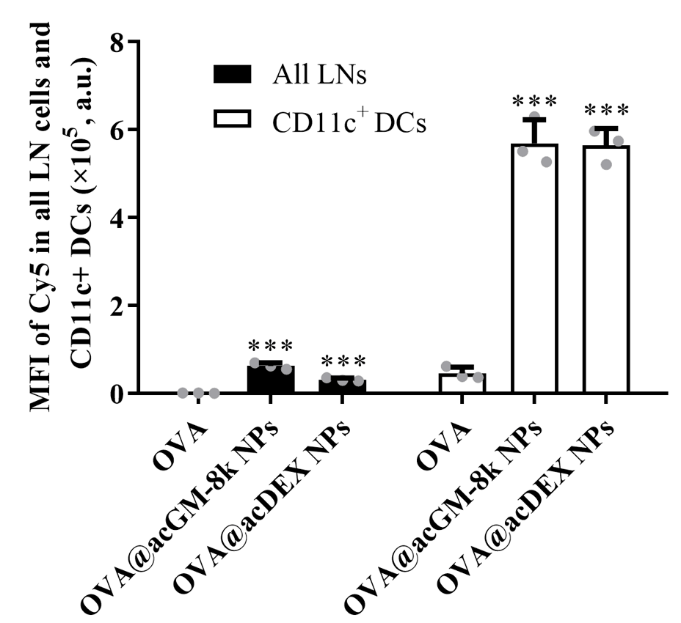


**Figure S4.** MFI of Cy5 in all LN cells and in CD11c^+^ DCs 36 h after footpad injection. **P* < 0.05, ** *P* < 0.01; ****P* < 0.001 vs. the OVA group; ns: no significance; *n* = 3: data were obtained from three mice. All numerical values are given as average values ± standard deviation. Statistical analysis was performed using Prism Software (GraphPad, USA), followed by one-way ANOVA analysis with Dunnett's post hoc evaluation.


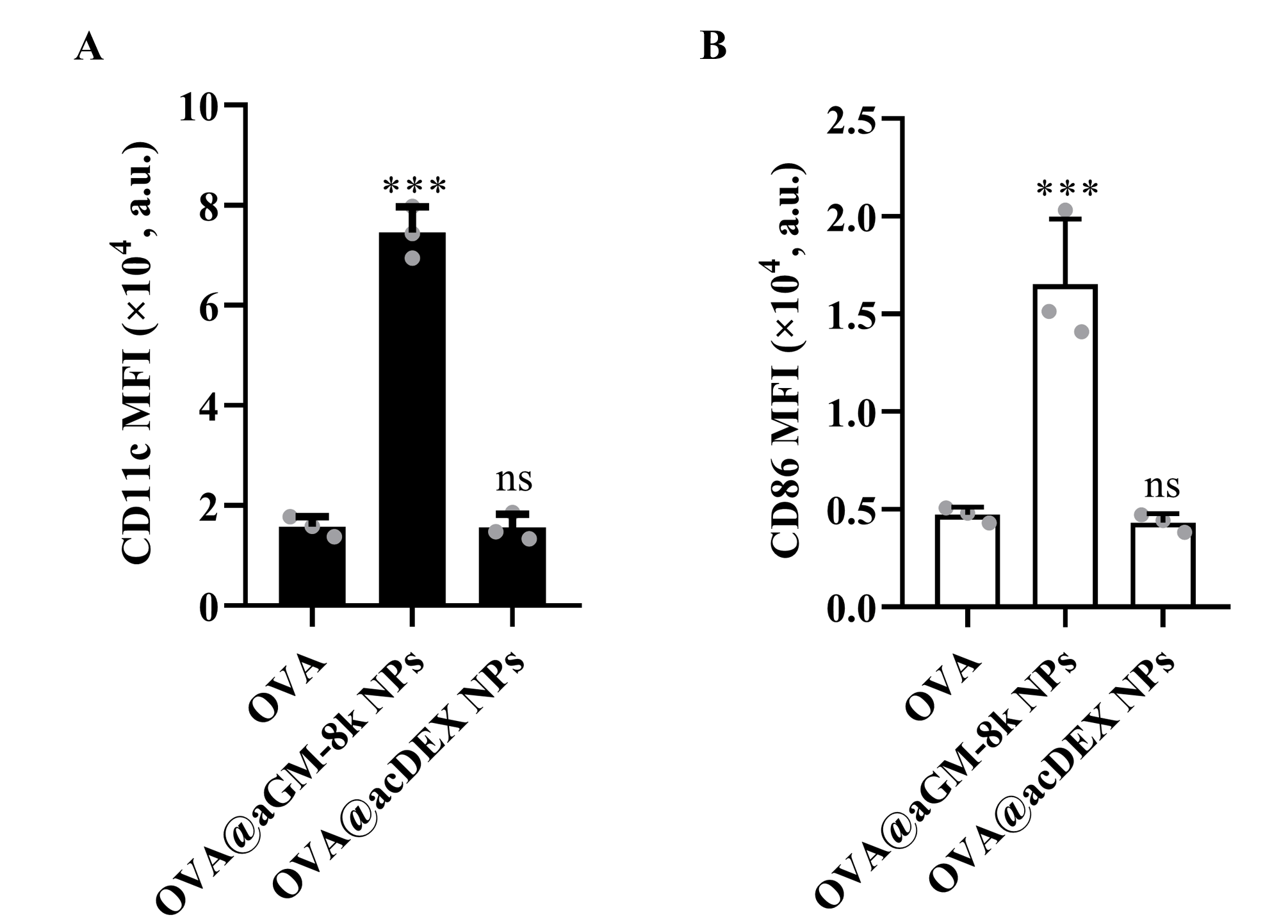


**Figure S5.** MFI of CD11c in LNs cells and CD86 in CD11c^+^ cells of LNs 72 h after immunization. (A) MFI of CD11c in LNs 72 h after immunization. (B) MFI of CD86 in CD11c^+^ cells 72 h after immunization. **P* < 0.05, ** *P* < 0.01; ****P* < 0.001 vs. the OVA group; ns: no significance; *n* = 3; Data were obtained from 3 mice. All numerical values are given as average values ± standard deviation. Statistical analysis was performed using Prism Software (GraphPad, USA), followed by one-way ANOVA analysis with Dunnett's post hoc evaluation.
